# Sustainable production of *Saussurea costus* under different levels of nitrogen, phosphorus and potassium fertilizers in cold desert region of Western Himalaya

**DOI:** 10.3389/fpls.2023.1179183

**Published:** 2023-06-20

**Authors:** Sakshi Vishvamitera, Diksha Dhiman, Sidharth Baghla, Satbeer Singh, Manish Kumar, Ashok Kumar, Dinesh Kumar, Sanatsujat Singh, Ramesh Chauhan

**Affiliations:** ^1^ Agrotechnology Division, Council of Scientific and Industrial Research - Institute of Himalayan Bioresource Technology, Palampur, India; ^2^ Chemical Technology Division, Council of Scientific and Industrial Research - Institute of Himalayan Bioresource Technology, Palampur, India

**Keywords:** chemical compound, critically endangered, dry root yield, essential oil, plant nutrition

## Abstract

**Introduction:**

*Saussurea costus*, an important critically endangered medicinal herb native to the Himalayan region, is commonly used in various ailments, *viz*. asthma, ulcer, inflammation, and stomach problems. In the international market, the dry roots and essential oil of *S. costus* has become an important drug. The lack of appropriate fertilizer dose recommendations is one of the limiting factors for its *ex-situ* conservation and large-scale cultivation, as plant nutrition is vital in determining crop growth and productivity. The study aimed to understand the comparative impact of different levels of fertilizer nutrients on growth, dry root and essential oil yield, and essential oil profile of *S. costus*.

**Methods:**

A field experiment was conducted in Himachal Pradesh, India's cold desert region (Lahaul valley), during 2020-21. The experiment comprised three levels of nitrogen (60, 90, and 120 kg ha^-1^), three levels of phosphorus (20, 40, 60 kg ha^-1^), and two levels of potassium (20 and 40 kg ha^-1^) in a factorial randomized block design.

**Results:**

The fertilizer application had an immense effect on growth attributes, root yield attributes, dry root yield, and essential oil yield over control. The treatment combination N120, P60, and K_40_ had the largest effect on the plant height, number of leaves per plant, leaf length and width, root length and diameter, dry matter per plant, dry root yield, and essential oil yield. However, the results were at par with the treatment comprising N_90_, P_40_, and K_20_. Dry root yield increased by 108.9%, and essential oil yield increased by 210.3% with fertilizer applications over unfertilized plots. The regression curve shows an increasing trend in dry root yield till N_90_, P_40_, and K_20_; after that, it nearly stabilized. The heat map showed that applying fertilizer significantly affected the chemical constituents of *S. costus* essential oil. Similarly, the plots fertilized with the highest level of NPK recorded the utmost value of available N, P, and K, as compared to unfertilized plots.

**Discussion:**

The results emphasize that for sustainable cultivation of *S. costus*, the application of N_90_, P_40_, and K_20_ combinations is the most suitable one.

## Graphical Abstract

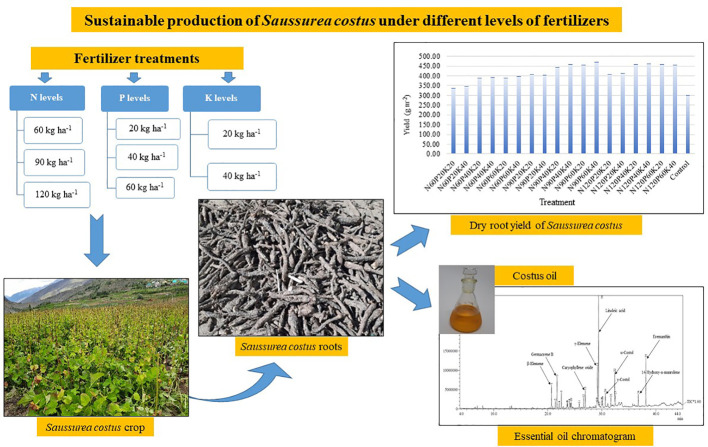

## 1 Introduction

Herbal drugs have recently received enormous popularity owing to their traditional and cultural causes among consumers. Approximately three-fourths of the global population relies heavily on plant species for curing health problems leading to pervasive growth in herbal-based pharmaceutical industries. Nearly 90% of the medicinal species are collected from wild sources, majorly (about 70%) through destructive harvesting (Sen and Chakraborty, 2017). The global estimated market for herbal medicines is nearly $2438.15 US, out of which India accounts for roughly 1% ($24.38 US) (Dhar and Dey, 2019). According to World Health Organization statistics, the global market worth of herbal products is anticipated to reach USD 5 trillion by 2050 (Shirolkar et al., 2015; Tahir et al., 2022) The Indian Himalayan region is a hotspot for herbal biodiversity and harbors more than 1748 plant species with known medicinal importance. One such genus of the family Asteraceae, with therapeutic benefits, is *Saussurea*, found in the wild at an elevation range of 2000-3500 meters above mean sea level (msl) in sub-alpine areas of the North-western Himalayas. Among all the species of *Saussurea*, *Saussurea costus* (Falc.) Lipsch. (Syn. *Saussurea lappa* C.B. Clarke), traditionally known as “Kuth” or “Kusth,” is the utmost economically viable species. Its therapeutic properties are exquisitely catalogued in Ayurvedic, Tibetan, and Chinese medicine systems. It is one of the essential constituents in about 175 herbal preparations documented in “The Handbook of Traditional Tibetan Drugs (Butola and Samant, 2010). Musky scented roots are the most valuable portion of the plant and are renowned for their medicinal properties with antifungal, anthelmintic, antidiabetic, antimicrobial, immuno-stimulant, antiulcer, anti-inflammatory and antihepatotoxic characteristics that make it one of the fascinating herbs on the planet (Shah, 2019). The essential oil (also known as costus oil) obtained from kuth roots is light yellow to brownish, resembling oriss oil to some extent. It is an important and costly drug in the International market. *S. costus* oil is used in high-grade perfume preparations, hair oil, insect repellent, and incense, owing to its blending capability with rose, sandalwood, patchouli, violet, vetiver, etc. (Rathore et al., 2020). The phytochemical investigation revealed that *S. costus* roots contain several bioactive components such as flavonoids, steroids, monoterpenes, lignans, sesquiterpenoids, flavonoids, glycosides, triterpenes, etc. (Benedetto et al., 2019). In addition, *S. costus* oil also possesses sesquiterpene lactones *viz.*, costunolide and dehydrocostus lactone, which exhibit anti-cancerous activities (Zahara et al., 2014). Fluctuations in environmental conditions, chemotype, phenophase and ecotype impart significant variations in essential oil composition of *S. costus*.

*S. costus* is classified as a critically endangered herbal plant and is nearly on the brink of extinction, the reason being its overexploitation from the wild (more than 90% from natural habitats) (Sen and Chakraborty, 2017). It is one of 24 herbal plant species that the National Medical Plants Board (NMPB) and Planning Commission, Government of India, have prioritized for research and development for *in-situ* as well as *ex-situ* conservation to achieve the intended goal of the medicinal plant sector (Kala et al., 2006). To bring it out from the “critically endangered status,” considering its therapeutic and commercial utility, farmers can play a pivotal role in conserving the herbal plant species through captive cultivation. Some innovative farmers of Lahaul valley, a cold desert region of Himachal Pradesh, started its cultivation on a large scale during the early 1900s but later replaced it with other cash crops like pea and potato owing to its long cultivation cycle and lower yield resulting in meager profits (Rawat et al., 2004; Ali and Venkatesalu, 2022). Despite government efforts to encourage the farmers for its cultivation, the response has not been very positive due to a lack of appropriate agro-technologies to attain higher yields with high marker compound content. The yield, quality, and composition of the secondary metabolites in medicinal plants depend on various biotic and abiotic factors. Among abiotic factors, soil fertility plays a significant role in developing quality medicinal plants (Verma and Shukla, 2015; Soltanbeigi, 2020). The quality and yield of herbal plants can be increased effectively by fertilization as amino acids and enzymes involved in the biosynthesis of numerous essential oil compounds are, in turn, synthesized by the plant using nitrogen (N), phosphorus (P), and potassium (K) primarily (Nurzynska-Wierdak, 2013).

The *S. Costus* plant has evidenced massive interest from several researchers in the recent past; still, there is a dearth of information on soil fertilization’s effect on its growth, yield, and marker compound content. Thus, it is imperative to establish standardized fertilizer practices to promote *S. costus* as an economically-attractive crop competitive with food crops and to improve the essential oil yield and marker compound content for its sustainable use in the herbal industry. This prompted us to comprehensively analyze the differences in growth, yield, and marker compounds of *S. costus* grown under varying dosages of N, P, and K to standardize the best fertilizer dosages for its large-scale cultivation.

## 2 Material and methods

### 2.1 Experimental site, agro-meteorological conditions, and soil properties

**Figure 1:**
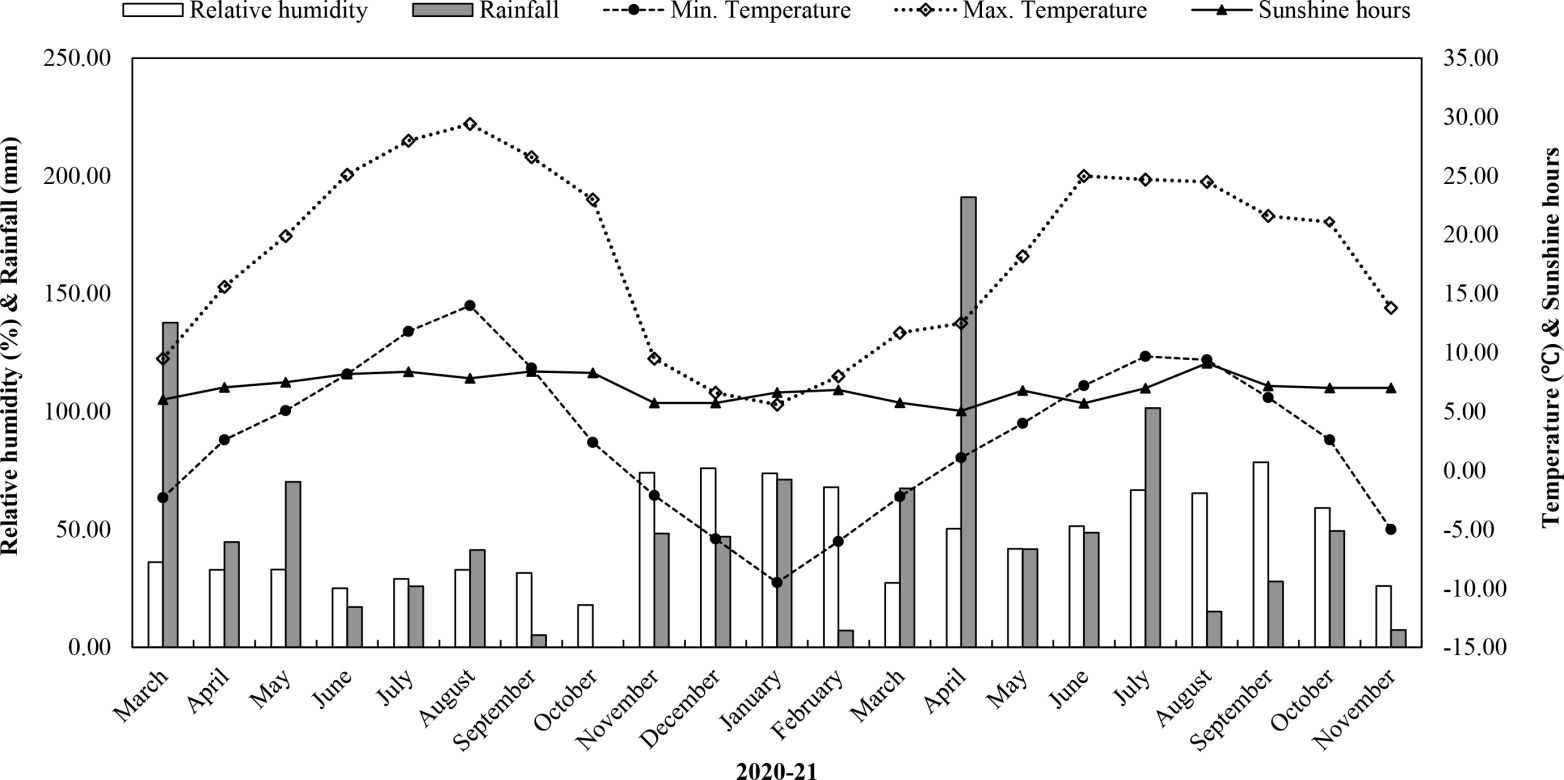
Average monthly meteorological data (relative humidity (%), rainfall (mm), mean maximum and minimum temperature (°C), and sunshine hours) during the cropping seasons of 2020 and 2021 at Lote, Distt. Lahaul & Spiti, Himachal Pradesh, India.

A field experiment was carried out to standardize the fertilizer dose for *S. costus *in Lahaul valley to attain higher root and essential oil yield in the growing season of 2020 and 2021. The experimental field is situated in the cold desert region of western Himalaya at Lote, Distt. Lahaul & Spiti, Himachal Pradesh, India (32035’42” N; 76056’36” E; 1393 m above msl). The study of the research site with respect to cropping pattern revealed that cauliflower was grown from May to August, then left fallow over the winter (only one crop season due to heavy snowfall during winter). Average meteorological data of two experimental years (2020 and 2021) is shown in **Figure 1**. The experimental location is characterized by heavy snowfall (733.90 mm) from November to May and scanty rainfall (332.40 mm) from June to October. The minimum and maximum temperature were -10°C and 29°C, respectively, with an average annual temperature of 10°C during both years. Before the experiment’s commencement, the research site’s soil sample was collected from 0-0.15 m depth to analyse the initial physico-chemical properties. The soil texture was sandy clay loam with pH 6.15. The soil available nutrient content was 382.40, 39.20, and 142.15 kg ha^-1^ of available N, P, and K, respectively. The organic carbon (OC) content in the soil of the research site was 0.67%.

### 2.2 Planting material and experimental specifications

One-year-old healthy seedlings of *S. costus *with 4-5 cm root length were obtained from the Plant Breeding section of Agrotechnology Division, CSIR- Institute of Himalayan Bioresource Technology, Palampur and transplanted in the field on 15^th^ May 2020. The experiment was laid out using a factorial randomized block design (FRBD) with three factors arrangement *i.e.*, three levels of N (60, 90, and 120 kg ha^-1^), three levels of P (20, 40, and 60 kg ha^-1^), two levels of K (20 and 40 kg ha^-1^) and control (where no fertilizer additions were made). The total treatment combinations (18) and one control were replicated thrice. The size of the experimental plots was 5 m^2^ (2.5 × 2.0 m) and seedlings were transplanted in square shape planting geometry with a spacing of 0.25 × 0.25 m. At the time of transplanting, one-third dose of N and the full dose of P and K were mixed thoroughly in soil, while the remaining two-third dose of N was supplied into two equal splits *i.e.*,30 and 60 DAT (days after transplanting). In the second growing season, P and K were mixed in the soil with the help of a hand hoe in May, while N was applied as per the preceding year. The nutrient fertilizer source was urea (46% N) for N, single super phosphate (16% P_2_O_5_) for P, and muriate of potash (60% K_2_O) for K. Weeding, irrigation, and other intercultural operations were performed as per the requirement of crop.

### 2.3 Sampling, measurements and methods

The impact of fertilizers treatment on plant growth and yield attributes was assessed by measuring the plant height, number of leaves per plant, leaf length & width, dry matter per plant, root length & diameter, and dry root yield. Ten plants were selected randomly from each plot to record all the growth parameters. Plant height at the harvesting stage was gauged from the ground level to the plant tip, whereas the number of leaves per plant was counted at the peak flowering period. The leaf length and width were determined by using the fresh leaves of selected plants with the help of a ruler and the mean value was calculated. For dry matter accumulation, the aerial part of selected plants was cut from the ground level using a sickle, and oven dried at 70 ± 2°C until a constant weight was achieved and averaged to analyze dry matter per plant. The root length was calculated by using intact roots of selected plants from each plot that were gently washed with running water to remove the soil, and then, root length was directly measured using a ruler, whereas root diameter was measured using a vernier calliper. For dry root yield estimation, ten plants’ scale and soil-free roots were dried under shade until a constant weight was obtained, and then the yield of ten plants was converted into yield per m^2^. After harvesting *S. costus*, the soil samples were collected from the top 0.15 m layer from each plot. The soil pH was calculated using pH meter (soil: water suspension, 1:2 w/v), whereas, Walkley and Black (1934) method was used to determine OC. Soil available N, P, and K were measured as per the method given by Subbiah and Asija (1956); Bray and Kurtz (1945), and Mehlich (1984), respectively.

### 2.4 Essential oil extraction

The essential oil was extracted from the dry roots of *S. costus* in three replicates as per treatment through hydrodistillation in a Clevenger apparatus. For that, 1 kg sample was placed in the distillation apparatus with 2 litres of water (1:2 ratio) and hydro-distilled for 6 hours. The obtained essential oil was dried by anhydrous sodium sulphate (Na_2_SO_4_) and kept separated in a sealed vial at 4°C for further examination under GC-MS and GC for identifying and quantifying the volatile compounds, respectively. The extracted essential oil content was computed as the weight of essential oil per weight of the sample (g per kg) on a dry weight basis, and essential oil yield was calculated by multiplying the dry root yield with essential oil content and expressed in kg per ha.

### 2.5 Gas chromatography analysis

The essential oil was analyzed with Shimadzu GC 2010 gas chromatograph with a flame ionization detector (FID). The SH-RX-5Si/MS capillary column, Shimadzu Asia pacific (USA) having dimensions of 30 m × 0.25 mm × 0.25 μm, was accoutred with gas chromatography. The auto-injection mode was run with a volume of 2 μL from 4 μL oil in 1.5 ml of CH_2_Cl_2_. Nitrogen was the carrier gas having a consistent velocity (1.05 ml min^-1^). The oven temperature was set to 70°C for 3 min., and then to 220°C for 5 min. at a constant rate (4°C per min). The detector and injector temperatures were 250 and 220°C, respectively. The initial pressure was nearly about 65.3 kPa, and the approximate linear velocity was 37.6 cm s^-1^.

### 2.6 Gas chromatography-mass spectrometry analysis

The concentrated essential oil samples were analyzed using Schimadzu GCMS-QP2010 (Shimadzu Corporation, Japan) system. An SH-RX-5Si/MS (30 m × 0.25 mm × 0.25 µm film thickness) column, fused with silica capillary was used, with nitrogen as a carrier gas at a flow rate of 1mL min^-1^. The injector temperature was set to 250°C, and the sample volume injected was 2µL. The oven temperature was programmed for 3.0 min. to hold at 70°C and then increased from 4°C to 220°C for 5.0 min, respectively. The ion source and interface temperature was 200°C, and 250°C, respectively, while, electron energy was 0.85eV. Retention indices (RI) of the compounds relative to a mixture of n-Alkanes (C_9_-C_24_) were determined.

### 2.7 Statistical analysis

The data collected on growth, yield parameters and chemical compound of *S. costus* were analyzed by the standard statistical analysis of variance (ANOVA) technique for FRBD. Fisher’s least significant difference (LSD) post-hoc test was performed to assess the differences among the treatments at 5% probability level (*P*=0.05). The multivariate principal component analysis (PCA), using the PAST 3 software, was used to understand the influence of treatments on growth, yield attributes and components of essential oil. The heat map was generated to assess the effect of treatments on composition of *S. costus* essential oil, and correlation studies were carried out to explore the relationship between different agronomic traits and essential oil compounds using R software. A first-degree regression model was established between fertilizer levels of N, P, and K and the dry root yield of *S. costus*.

## 3 Results and discussion

### 3.1 ANOVA due to different treatments

ANOVA showed that levels of N, P and K significantly affected the number of leaves per plant, dry matter per plant and root diameter of *S. costus* (**Table 1**). The leaf length, leaf width and root length were found significant only for N and P, while the plant height was significant for N and K. For dry root yield, the individual effect of N, P and K was found significant; however, their interaction effect was non-significant. None of the treatments (N, P and K) and their interaction (N×P, N×K, P×K and N×P×K) was found significant in case of essential oil content, while the sole effect of N, P and K was found significant for essential oil yield. The interaction effect of N and P (N×P) was found significant for plant height, number of leaves per plant, leaf width, dry matter per plant, root length and diameter; however, other interactions were found non-significant for all the growth and yield attributes, dry root and essential oil yield and essential oil content. When compared with control, all the treatments (N, P and K) were found significant for all the growth and yield attributes, dry root and essential oil yield, and essential oil content.

**Table 1:** Analysis of variance for the effects of N, P, and K levels on no. of leaves per plant, leaf length & width, dry matter per plant, root length & diameter, dry root yield, essential oil content, and essential oil yield of *S. costus*.

Source of variation	Degree of freedom	Plant height	No. of leaves plant^-1^	Leaf length	Leaf width	Dry matter plant^-1^	Root length	Root diameter	Dry root yield	Essential oil content	Essential oil yield
Treatment	18	366.57*	1.72*	4.55*	6.25*	4.35*	36.78*	94.78*	6931.35*	0.083*	6.13*
N	2	1738.69*	6.01*	15.89*	25.29*	17.95*	170.19*	380.04*	26123.74*	0.013	14.92*
P	2	40.86	2.54*	5.26*	11.04*	7.70*	70.11*	152.46*	15622.19*	0.006	8.42*
K	1	189.48*	0.44*	2.50	0.33	0.88*	2.34	4.92*	471.93*	0.008	0.69*
N×P	4	121.99*	0.95*	0.24	1.61*	1.54*	11.60*	3.93*	88.64	0.004	0.09
N×K	2	98.94	0.06	0.79	0.90	0.01	0.17	0.27	73.61	0.003	0.17
P×K	2	4.81	0.15	1.25	0.29	0.13	0.55	0.15	4.04	0.004	0.10
N×P×K	4	52.52	0.09	0.79	0.04	0.22	1.58	0.21	132.66	0.005	0.15
Control vs others	1	1944.06*	8.83*	29.00*	30.57*	18.89*	124.96*	618.67*	39760.02*	1.393*	61.52*
Error	36	35.02	0.10	0.74	0.38	1.56	1.87	1.01	107.96	0.006	0.14

Values represent the mean sum of squares.

*Significant at 0.05 levels of probability.

### 3.2 Growth attributes

The analyzed data presented in **Table 2** revealed that different growth parameters, *viz.*, plant height at harvest, number of leaves per plant, leaf length & width, were significantly affected by varying levels of N. The highest level of N, *i.e.*, N_120_, produced the tallest plants of *S. costus* (181.39 cm), which is significantly (*P*<0.05) different from N_60_ and N_90_, however, the percent increase in plant height from N_60_ to N_90_ was higher, almost two times, compared from N_90_ to N_120_. On comparing different P levels, higher plant height (173.75 cm) was observed with P_40_; however, no significant differences were observed among varying P levels. Under different K levels, the tallest plants (174.55 cm) were registered with the highest level of K, *i.e.*, K_40_, which was significantly different from K_20_. In comparison, the control plots without adding NPK recorded the shortest plants (146.52 cm). An increase in N levels significantly augmented the height of plant that could be resulted from the beneficial effect of N in stimulating the meristematic activity rapidly for developing more tissues and organs as well as its essential role in protein synthesis, in addition to its vital role in several biochemical processes related to plant height. This corroborates the results of Eleiwa et al. (2012) in wheat (*Triticum aestivum*). The other two macronutrients (P and K) play an essential role in increasing the length and number of internodes leading to a progressive increment in plant growth with their increasing levels. The findings are in accordance with the results of Kwon et al. (2019) in bellflower (*Platycodon grandiflorum*), an important medicinal plant, and Arab et al. (2015) in marigold (*Calendula officinalis*).

**Table 2:** Effect of N, P, and K levels on plant height, no. of leaves per plant, leaf length & width, dry matter per plant, root length & diameter, dry root yield, essential oil content, and essential oil yield of *S. costus*.

Treatments	Plant height (cm)	No. of leaves plant^-1^	Leaf length (cm)	Leaf width (cm)	Dry matter plant^-1^ (g)	Root length (cm)	Root diameter (mm)	Dry root yield (g m^-2^)	Essential oil content (%)	Essential oil yield (kg ha^-1^)
Nitrogen Level (kg ha^-1^)										
N_60_	162.03^c^	6.31^b^	15.85^c^	12.98^b^	7.81^b^	20.44^c^	37.69^b^	375.31^b^	2.12	7.95^b^
N_90_	174.61^b^	7.20^a^	17.03^b^	14.80^a^	9.43^a^	25.20^b^	45.32^a^	440.19^a^	2.14	9.44^a^
N_120_	181.39^a^	7.39^a^	17.71^a^	15.21^a^	9.64^a^	26.19^a^	45.94^a^	442.34^a^	2.17	9.61^a^
SEm(±)	1.39	0.08	0.20	0.15	0.10	0.32	0.24	2.45	0.02	0.09
LSD (*P* = 0.05)	4.00	0.22	0.58	0.42	0.30	0.92	0.68	7.02	NS	0.26
Phosphorus Level (kg ha^-1^)										
P_20_	170.95	6.54^b^	16.31^b^	13.50^c^	8.21^b^	21.73^c^	39.65^c^	385.35^b^	2.13	8.22^b^
P_40_	173.75	7.16^a^	16.89^ab^	14.43^b^	9.31^a^	24.58^b^	44.26^b^	434.09^a^	2.14	9.28^a^
P_60_	173.33	7.21^a^	17.39^a^	15.06^a^	9.37^a^	25.52^a^	45.04^a^	438.39^a^	2.17	9.50^a^
SEm(±)	1.39	0.08	0.20	0.15	0.10	0.32	0.24	2.45	0.02	0.09
LSD (*P* = 0.05)	NS	0.22	0.58	0.42	0.30	0.92	0.68	7.02	NS	0.26
Potassium Level (kg ha^-1^)										
K_20_	170.80^b^	6.88^b^	16.65	14.25	8.83^b^	23.73	42.68^b^	416.32^b^	2.13	8.89^b^
K_40_	174.55^a^	7.06^a^	17.08	14.41	9.09^a^	24.15	43.29^a^	422.23^a^	2.16	9.11^a^
SEm(±)	1.14	0.06	0.17	0.12	0.08	0.26	0.19	2.00	0.01	0.07
LSD (*P* = 0.05)	3.27	0.18	NS	NS	0.24	NS	0.55	5.74	NS	0.21
Control	146.52^b^	5.21^b^	13.67^b^	11.05^b^	6.38^b^	17.31^b^	28.23^b^	301.00^b^	1.44^b^	4.35^b^
Others	259.02^a^	10.45^a^	25.30^a^	21.49^a^	13.44^a^	35.91^a^	64.48^a^	628.92^a^	3.22^a^	13.50^a^
SEm(±)	2.48	0.14	0.36	0.26	0.18	0.57	0.42	4.36	0.03	0.16
LSD (*P* = 0.05)	7.12	0.39	1.04	0.74	0.53	1.65	1.21	12.50	0.09	0.46

SEm indicates Standard Error of Mean; LSD indicates Least Significant Difference; NS indicates not significant at P = 0.05.

The meaning of lowercase letter is N60, N90, N120, represents N levels @ 60, 90, and, 120 kg ha-1, respectively; P20, P40 and P60, represents P levels @ 20, 40 and 60 kg ha-1, respectively; and K20 and K40 indicates K levels @ 20 and 40 kg ha-1, respectively.

Applying different levels of N significantly influenced the number of leaves per plant. The highest value *i.e.* 7.39 was found in the plots fertilized with the highest N level (N_120_), which was statistically at par (7.20) with N_90_; however, both N_90_ and N_120_ levels were significantly different from N_60_. Different levels of P and K also significantly affected the number of leaves per plant. The effect of P_40_ and P_60_ was significantly higher compared with P_20_; however, these two levels were at par with each other, and the highest number of leaves per plant (7.21) was registered with the highest level of P, *i.e.*, P_60_. A similar pattern was noticed in the case of K, where the highest level of K (K_40_) resulted in a higher number of leaves per plant (7.06); however, it did not produce any significant differences with K_20_. Moreover, absolute control without fertilizer addition registered the lowermost number of leaves per plant. Higher NPK fertilizer doses might result in maintaining optimal cytokinin-auxin ratio by promoting more activity of cytokinin in roots, which leads to activate the dormant axillary buds into branches, thereby, resulting in higher number of leaves per plant. Furthermore, plant height was significantly affected by higher doses of N, P, and K, which might contribute to a higher number of leaves per plant with augmented levels of macroelements. The results are in agreement with the findings of Kawatani et al. (1980); Aladakatti et al. (2012) and Mahajan et al. (2021) in *Stevia rebaudiana*.

The performance of *S. costus* plant in terms of leaf length and width was recorded as superior (17.71 cm and 15.21 cm, respectively) with N_120_; however, in the case of leaf width, N_120_ was found statistically at par with N_90_. When different P levels were compared, the highest leaf length (17.39 cm) and width (15.06 cm) were registered in P_60_, which produced non-significant (*P*<0.05) differences with P_40_ (16.89 cm and 14.43 cm, respectively). Nevertheless, the increasing scale from P_20_ to P_40_ was higher than from P_40_ to P_60_. Among different K levels, K applied @ 40 kg ha^-1^ (K_40_) recorded the highest leaf length (17.08 cm) and width (14.41 cm); though changes among levels of K were found non-significant. The positive impact of higher levels of N on leaf growth could be attributed to its essential role in chlorophyll, enzymes, and protein synthesis, which tends to increase the length and width of the leaves. In the current study, applying higher doses of P leads to considerably higher leaf length and width that might be attributed that P is a vital constituent of several essential molecules such as phospholipids, ATP, and nucleic acids for energy transfer, protein synthesis, carbohydrate, and lipid metabolism (Rhykerd and Overdahl, 1972). The results conform to the findings of Medda and Hore (2003); Mohan et al. (2004) and Verma et al. (2019) in *Curcuma longa*. The application of fertilizers causing a significant increase in all the growth attributes over control could be resulted from the adequate provision of essential nutrients to the plants.

### 3.3 Dry matter per plant

The analyzed data (**Table 2**) revealed that the dry matter per plant was superior with N_120_ (9.64_ g_); however, it produced non-significant differences with N_90_ (9.43_ g_). Irrespective of P and K treatments, N_120_ significantly (*P*<0.05) increased the dry matter per plant by about 23% compared with N_60_. Different levels of P also significantly affected the dry matter per plant, and the plants fertilized with P_60_ produced the highest dry matter per plant (9.37 g), though it remained statistically at par (9.31 g) with P_40_. Despite that, the increase in dry matter per plant from P_20_ to P_40_ was higher than from P_40_ to P_60_. The consequences of applied K on dry matter per plant were found to be significant, and the utmost value (9.09 g) was attained with K_40_. In addition, the lowest dry matter per plant was recorded under control (6.38 g).

A positive effect of higher levels of nutrients on the number of leaves per plant, leaf length & width due to taller plants might have resulted in increased dry matter per plant with increasing levels of nutrient fertilizers. The results of Chalapathi et al. (1999) and Aladakatti et al. (2012) in *Stevia rebaudiana*, supported the findings of the present study.

### 3.4 Root yield attributes and dry root yield

The data given in **Table 2** described that the root length and diameter were significantly (*P*<0.05) influenced by varying levels of N. The maximum root length (26.19 cm) was recorded with N_120_; however, the increase from N_60_ to N_90_ was higher than N_90_ to N_120_. Root length was also significantly affected by levels of P and K, and the highest root length, *i.e.*, 25.52 cm and 24.15 cm was recorded with P_60_ and K_40_, respectively, though, the differences in root length with different levels of K were found non-significant.

Root diameter showed significant variations with different levels of N and the highest root diameter (45.94 mm) was registered with N_120_, which was at par (45.32 mm) with N_90_. Nevertheless, the percent increase in root diameter from N_60_ to N_90_ was about 21.9%, and from N_90_ to N_120_, it was 1.4%. Root diameter in response to P showed the utmost value (45.04 mm) with P_60_, which showed significant variations with P_20_ and P_40_. Irrespective of applying N and P, the root diameter of *S. costus* was significantly affected by varying levels of K, and the highest root diameter was found to be 43.29 mm with K_40_. In addition, the lowest root diameter (28.23 mm) was found in the control, where no fertilizer was applied.

In the present study, root length and diameter were significantly higher with higher doses of N, which could be attributed that N stimulates lateral root elongation and development through the production of auxin and cytokinin, respectively in response to a systematic N-signalling (Takei et al., 2001; Krouk et al., 2010). The positive impact of higher doses of P and K on root growth parameters might be due to the vital role of P in root morphology *via* affecting the carbon budget of the whole plant (Mollier and Pellerin, 1999), and furthermore, K has a pivotal role in the photosynthates’ translocation from source (leaves) to sink (roots) that led to increased root growth. Kwon et al. (2019) in their study on a medicinally important plant, *Platycodon grandiflorum, *also reported similar results.

The dry root yield in response to N was significant, and the highest dry root yield (442.34 g m^-2^) was recorded with N_120_, that showed significant variations with N_60_. The effect of N_120_ and N_90_ on dry root yield was significantly higher than that of N_60_; however, both treatments were statistically at par. The dry root yield of *S. costus* was also significantly affected by P and K, and the highest dry root yield *i.e.*, 438.39 and 422.23 g m^-2^ was registered with P_60_ and K_40_, respectively. In contrast, the lowest dry root yield was recorded under control. The rise in dry root yield with higher levels of fertilizers might be attributed to favorable impact of utmost levels of N, P, and K on root yield attributes like root length and diameter. The findings of the present study corroborate the earlier results of Singh and Negi (2003) in *S. costus*.

### 3.5 Essential oil content and essential oil yield

The analyzed data revealed that essential oil content was not considerably influenced by varying levels of N, P, and K fertilizers; however, it increased slightly with increased doses (**Table 2**). Nevertheless, the effect of fertilizer application on essential oil content was significantly (*P ≤* 0.05) higher when compared to the control. Averaged across P and K levels, essential oil yield showed significant variations with different levels of N, and highest essential oil yield (9.61 kg ha^-1^) was registered with N_120_, which was found at par (9.44 kg ha^-1^) with N_90_. However, the percent increase in essential oil yield from N_60_ to N_90_ was about 19%, and from N_90_ to N_120_, it was only 2%. The essential oil yield of *S. costus* in response to P was found to be significant, and the highest essential oil yield (9.50 kg ha^-1^) was found in P_60_, which showed non-significant variations with P_40_ (9.28 kg ha^-1^). Irrespective of applying N and P, varying levels of K fertilizer also significantly influenced the essential oil yield, and the maximum essential oil yield (9.11 kg ha^-1^) was recorded with K_40_, which showed significant variations with K_20_. In addition, overall fertilizer application (13.5 kg ha^-1^) recorded significantly higher essential oil yield over control (4.35 kg ha^-1^) where no NPK was applied.

The positive impact of higher NPK doses on essential oil yield might be attributed to their direct or indirect involvement in the production of primary or secondary metabolites, in addition to the favorable effect of macro-elements on root growth parameters and root yield. The results are in accordance with the earlier findings of Bhuvaneshwari et al. (2002) in *Pimpinella anisum*; Abbaszadeh and Haghighi (2013) in *Thymus vulgaris*.

### 3.6 Essential oil composition

**Figure 2:**
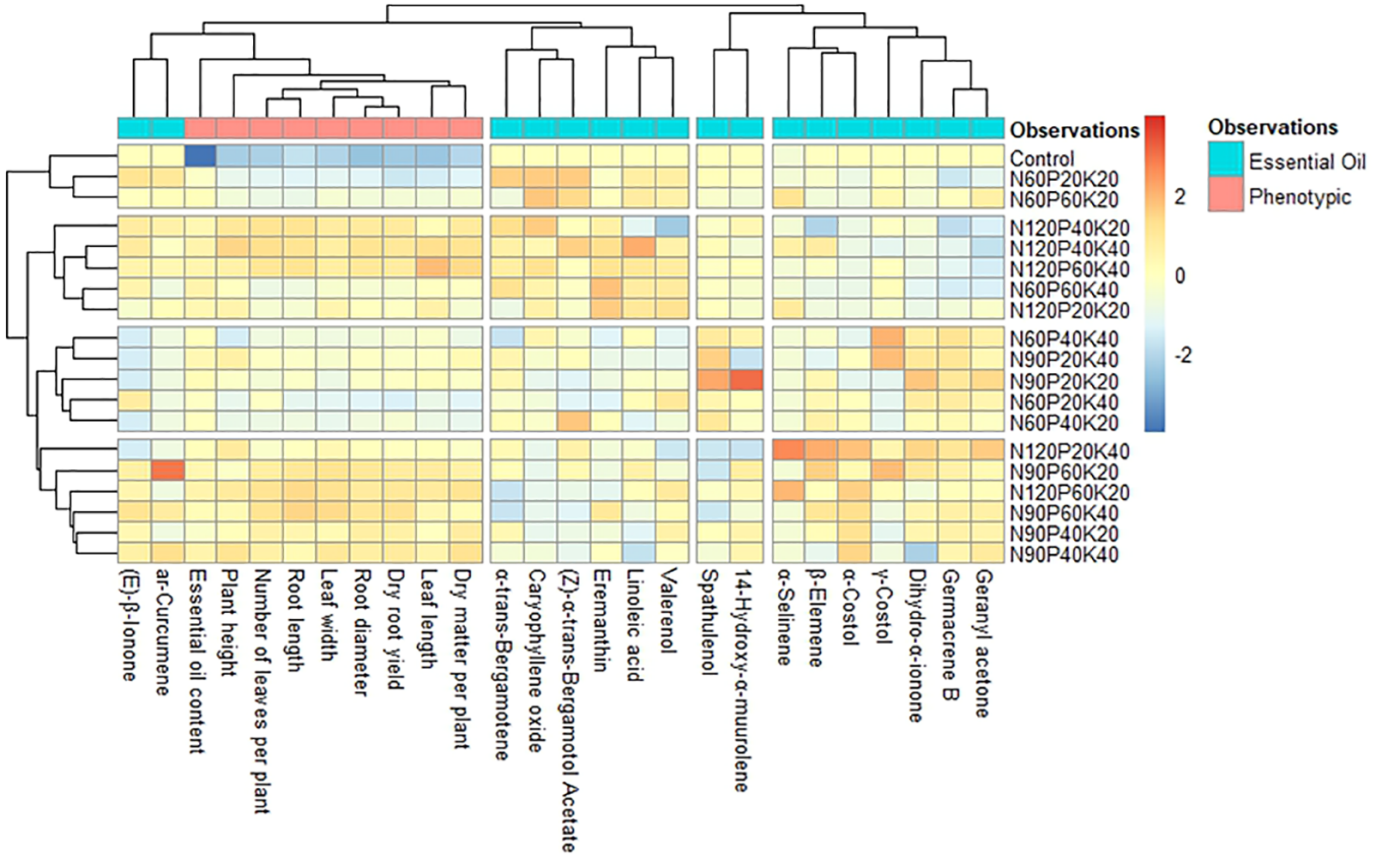
Heat map depicting the variation of chemical compounds identified in the costus oil in response to different NPK treatment combinations. N_60_, N_90_ and N_120_: N applied @ 60, 90 and 120 kg ha^-1^, respectively; P_20_, P_40_ and P_60_: P applied @ 20, 40 and 60 kg ha^-1^, respectively; K_20_ and K_40_: K applied @ 20 and 40 kg ha^-1^, respectively.

In the present study, a total of 25 compounds in the essential oil of *S. costus* were identified. Only 17 major compounds, which contributed about 57.66-78.63% were analyzed in the heat map that depicts the variations in the chemical profile of volatile constituent of essential oil owing to the interaction of varying levels of N, P, and K (**Figure 2**). Heat map analysis indicated that accumulation pattern of the chemical compound was not constant over the treatment combination. The clustering showed that N_90_P_20_K_20_ recorded a higher concentration of 14-hydroxy-α-muurolene, spathulenol and dihydro-α-ionone. However, the concentration of ar-curcumene and γ-costol were higher under N_90_P_60_K_20_. Similarly, application of N_120_P_20_K_40_ resulted in higher concentration of α-selinene, β-elemene and α-costol while, linoleic acid was recorded higher under N_120_P_40_K_40_.

**Figure 3:**
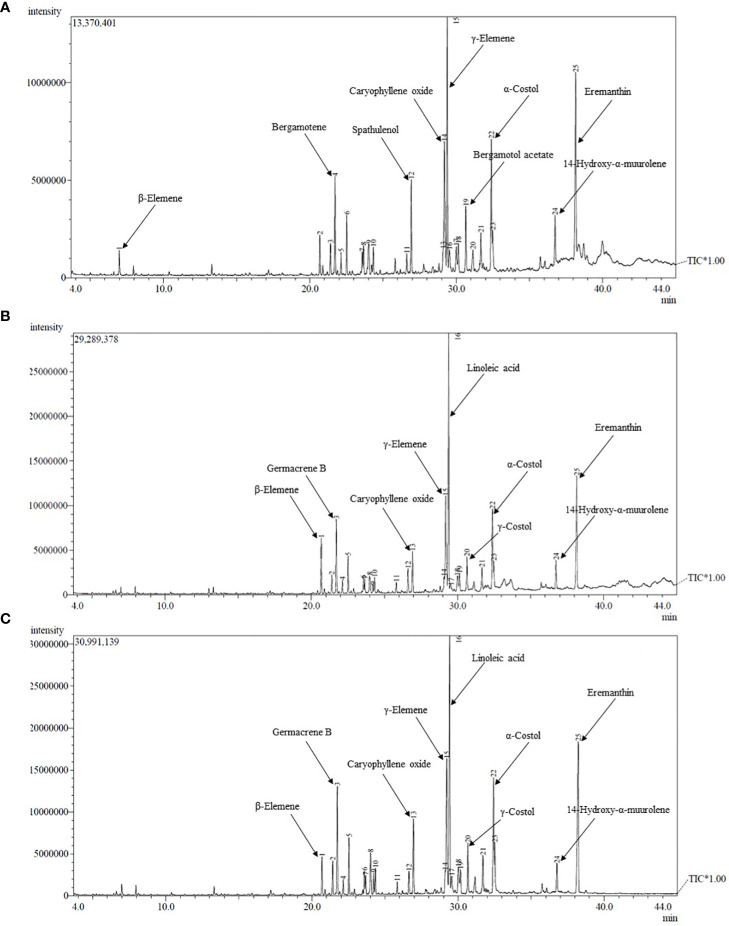
GC-MS Chromatogram of essential oil of *S. costus* in response to different treatment combinations; **(A)** Control, **(B)** N_90_P_40_K_20_ and **(C)** N_120_P_60_K_40_.

To understand the individual effect of N, P, and K levels on chemical compositions, their mean data is presented in **Table 3**. Means given in **Table 2** revealed that except β-elemene, all other chemical compounds were significantly affected by different levels of N, P and K, however the pattern of concentration was not uniform between the levels. The higher amount of N levels *i.e.* N_120_ resulted in significantly highest concentration of (E)-β-ionone, α-selinene, linoleic acid and eremanthin over N_90_ and N_60_, while α-trans-bergamotene was at par with N_60_. However, N_90_ recorded highest values for dihydro-α-ionone, germacrene B, geranyl acetone, ar-curcumene, α-costol and 14-hydroxy-α-muurolene. The other compounds *viz.* spathulenol, caryophyllene oxide, (Z)-α-trans-bergamotol acetate and valerenol were highest under N_60_; and γ-costol was at par with N_90_. Among P levels, P_60_ resulted in significantly highest concentration of (E)-β- ionone, ar-curcumene, linoleic acid, γ-costol, valerenol, α-costol and eremanthin. Similarly, P_40_ recorded maximum values for (Z)-α-trans-bergamotol acetate and 14-hydroxy-α-muurolene while caryophyllene oxide was at par with P_60_. The lowest level of P *i.e.* P_20_ resulted in highest concentration of dihydro-α-ionone, germacrene B, α-trans-bergamotene, geranyl acetone, α-selinene and spathulenol. Under K levels, K_40_ recorded highest concentration for germacrene B, linoleic acid, γ-costol, α-costol and eremanthin over K_20_. While the other chemical compounds *viz.* α-trans-bergamotene, ar-curcumene, spathulenol, caryophyllene oxide, (Z)-α-trans-bergamotol acetate, valerenol and 14-hydroxy-α-muurolene were recorded higher under K_20_. The increase in essential oil constituents with application of fertilizers might be resulted from improved availability of essential nutrients that consequently led to improved photosynthetic rate which affects the formation of precursor compounds of fatty acids that determines the oil quality, in addition to their role in development of glandular trichomes, secretory ducts and oil channels (Salehi et al., 2019; Chiyaneh et al., 2022). Further, several researchers also reported that the application of fertilizer nutrients alters the composition of essential oil in many medicinal and aromatic plants like *Artemisia dracunculus* (Heidari et al., 2014), *Rosa damascena* (Pal & Mahajan, 2017) and *Cymbopogon flexuosus* (Zheljazkov et al., 2011). The representative GC-MS chromatogram of *S. costus* essential oil in response to different treatment combinations are given in **Figure 3**.

**Table 3:** Variation in essential oil composition of *S. costus* due to N, P, and K levels.

Treatments	β-Elemene	Dihydro-α-ionone	Germacrene B	α-trans-Bergamotene	Geranyl acetone	(E)-β- Ionone	α- Selinene	ar-Curcumene	Spathulenol	Caryophyllene oxide	Linoleic acid	(Z)-α-trans-Bergamotol Acetate	γ-Costol	Valerenol	α-Costol	14-Hydroxy-α-muurolene	Eremanthin
RT	20.7	21.4	21.7	22.1	22.5	23.6	24.2	23.7	26.6	26.9	29.4	30.2	31.7	32.4	32.5	36.8	38.2
RI	1392	1413	1420	1433	1445	1477	1495	1479	1576	1585	1668	1700	1747	1772	1775	1934	1990
Nitrogen Level (kg ha^-1^)																	
N_60_	2.57	1.80^b^	4.14^b^	2.34^a^	2.33^b^	1.48^b^	0.42^b^	0.47^c^	1.60^a^	4.62^a^	17.17^b^	6.17^a^	3.88^a^	8.68^a^	2.13^c^	3.01^b^	6.47^b^
N_90_	2.70	2.05^a^	5.89^a^	2.07^b^	2.92^a^	1.54^b^	0.22^c^	1.26^a^	1.33^b^	0.51^c^	14.42^c^	2.12^c^	3.87^a^	7.2^4c^	6.62^a^	4.00^a^	5.87^c^
N_120_	2.64	1.62^c^	3.30^c^	2.39^a^	2.08^c^	1.71^a^	1.10^a^	0.70^b^	1.06^c^	3.84^b^	17.74^a^	5.02^b^	3.18^b^	7.62^b^	4.48^b^	2.63^c^	8.96^a^
SEm(±)	0.05	0.04	0.04	0.03	0.04	0.02	0.02	0.01	0.05	0.03	0.13	0.04	0.07	0.09	0.06	0.07	0.13
LSD	NS	0.10	0.13	0.09	0.11	0.06	0.06	0.03	0.15	0.08	0.36	0.13	0.20	0.26	0.18	0.20	0.37
Phosphorus Level (kg ha^-1^)																	
P_20_	2.68	2.17^a^	5.02^a^	2.63^a^	2.73^a^	1.07^c^	0.80^a^	0.47^c^	1.69^a^	2.58^b^	16.74^b^	4.16^b^	3.67^b^	7.88^b^	3.83^c^	2.98^c^	5.79^c^
P_40_	2.54	1.63^b^	4.30^b^	2.34^b^	2.29^b^	1.53^b^	0.30^c^	0.70^b^	1.51^b^	3.20^a^	14.91^c^	5.12^a^	2.55^c^	6.16^c^	4.55^b^	3.56^a^	7.18^b^
P_60_	2.70	1.68^b^	4.01^c^	1.83^c^	2.32^b^	2.14^a^	0.65^b^	1.26^a^	0.79^c^	3.19^a^	17.67^a^	4.04b	4.71^a^	9.49^a^	4.85^a^	3.09^b^	8.33^a^
SEm(±)	0.05	0.04	0.04	0.03	0.04	0.02	0.02	0.01	0.05	0.03	0.13	0.04	0.07	0.09	0.06	0.07	0.13
LSD	NS	0.10	0.13	0.09	0.11	0.06	0.06	0.03	0.15	0.08	0.36	0.13	0.20	0.26	0.18	0.20	0.37
Potassium Level (kg ha^-1^)																	
K_20_	2.61	1.86	4.10^b^	2.37^a^	2.45	1.58	0.60	0.99^a^	1.41^a^	3.35^a^	16.27^b^	5.10^a^	3.22^b^	7.99^a^	3.95^b^	4.02^a^	6.72^b^
K_40_	2.67	1.79	4.78^a^	2.16^b^	2.44	1.57	0.57	0.62^b^	1.25^b^	2.62^b^	16.61^a^	3.78^b^	4.06^a^	7.70^b^	4.87^a^	2.40^b^	7.48^a^
SEm(±)	0.04	0.03	0.04	0.02	0.03	0.02	0.02	0.01	0.04	0.02	0.10	0.04	0.06	0.08	0.05	0.06	0.10
LSD	NS	NS	0.11	0.07	NS	NS	NS	0.03	0.12	0.07	0.29	0.10	0.16	0.22	0.14	0.17	0.30
Control	2.64	1.83	4.44	2.27	2.44	1.58	0.07^b^	0.81	1.33	2.99	16.44	4.44	3.86	7.85	4.41	3.21	7.10
Others	3.96	2.74	6.66	3.40	3.67	2.37	0.87^a^	1.21	1.99	4.48	24.66	6.66	5.46	11.77	6.61	4.82	10.65
SEm(±)	0.09	0.06	0.08	0.05	0.07	0.04	0.04	0.02	0.09	0.05	0.22	0.08	0.12	0.16	0.11	0.13	0.23
LSD	NS	NS	NS	NS	NS	NS	0.11	NS	NS	NS	NS	NS	NS	NS	NS	NS	NS

RT-Retention time; RI-Retention indices.

N60, N90, N120, represents N levels @ 60, 90, and, 120 kg ha-1, respectively; P20, P40 and P60, represents P levels @ 20, 40 and 60 kg ha-1, respectively; and K20 and K40 indicates K levels @ 20 and 40 kg ha-1, respectively. NS indicates “not significant at P = 0.05”.

### 3.7 PCA

**Figure 4:**
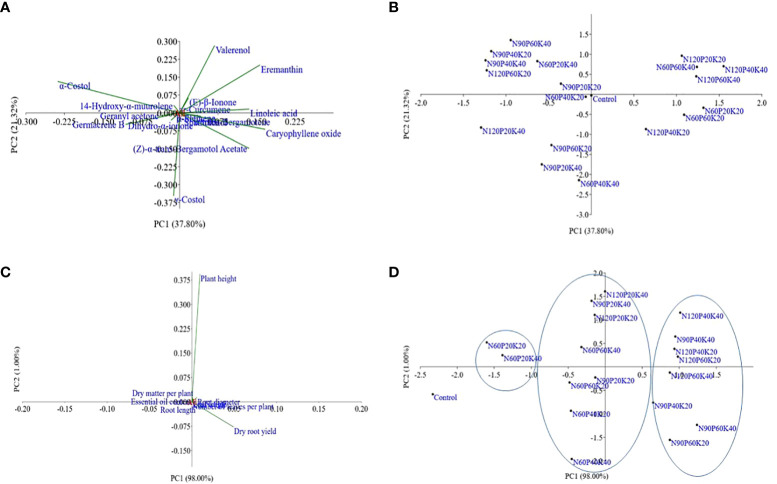
Scatter plots of principal components analysis **(A)** bi-plot of biochemical constituents **(B)** treatments’ distribution based on biochemical constituents, **(C)** bi-plot of agronomic traits, and **(D)** treatments distribution based on agronomic traits. In the bi-plots, N60, N90, N120, represents N levels @ 60, 90, and, 120 kg ha-1, respectively, P20, P40 and P60, represents P levels @ 20, 40 and 60 kg ha-1, respectively, and K20 and K40 indicates K levels @ 20 and 40 kg ha-1, respectively.

The PCA was carried out using 17 chemical compounds of essential oil isolated from dried roots of *S.* *costus*. The PCA analyzed data showed that component 1 and 2 (PC_1_ and PC_2_) jointly accounted for 59.12% of the total variations in biochemical traits (**Figures 4A, B**). The eigenvalues of PC_1_ and PC_2_, (the relevant and informative principal components), was 36.5 and 20.6, respectively. Component 1 (explained about 37.80% of the total variations), was found positively correlated with linoleic acid, caryophyllene oxide, (Z)-α-trans-bergamotol acetate, α-trans-bergamotene, ar-curcumene, eremanthin and spathulenol with loading values of 0.33, 0.41, 0.33, 0.10, 0.02, 0.38 and 0.02, respectively, and negatively correlated with rest of the compounds. In addition, the compound valerenol related more positively to PC_2_ having loading value of 0.52. The γ-costol was separated from the other compounds by PC_1_ and PC_2_ and situated in the negative coordinate. The PCA bi-plot did not show any specific trend for the treatment effects on major compounds of essential oil (**Figure 4B**).

PCA was also performed to study the interaction of different levels of N, P, and K on *S. costus’ *agronomic traits (**Figures 4C, D**). The findings of the PCA indicated that the first component solely contributed to 98.0% of the total variations in agronomic traits. Among all the agronomic traits, dry root yield contributed the maximum towards the total variation with a loading value of 0.99. The PCA bi-plot (**Figure 4D**) categorized the treatment combinations into three distinct clusters, and all the treatment combinations comprising N_60_ were positioned in the negative end of PC_1_. In addition, all the N_120_ consisting treatments were located in the positive end of PC_1_, except N_120_P_20_K_20_. PC_1_ also separated all the treatment combinations comprising P_20_, irrespective of N and K levels, and put them in the negative coordinate.

### 3.8 Correlation and regression analysis

**Figure 5:**
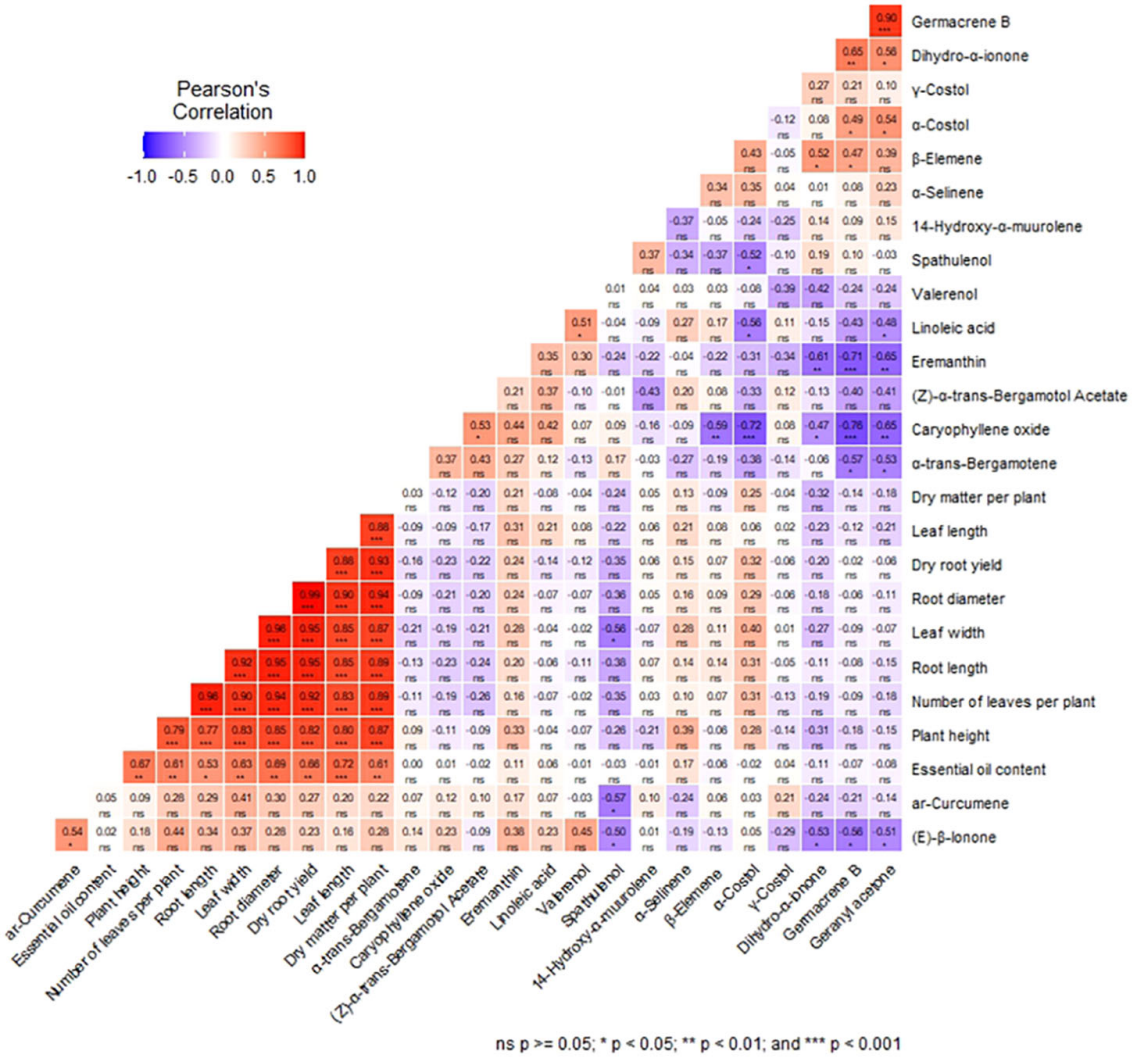
Correlation matrix among the agronomic traits, and 17 major compounds of costus oil. *, **, *** indicates significant at P = 0.05, P = 0.01 and P = 0.001, respectively‘ns’ indicates not significant at P = 0.05.

The correlation matrix among agronomic traits (plant height, number of leaves per plant, leaf length & width, root length & diameter, dry matter per plant, dry root yield), and essential oil components of *S. costus* is depicted in **Figure 5**. The figure showed that all the agronomic traits exhibited a strongly positive correlation with each other with r values >0.77 (p<0.001). The robust relationship between dry root yield, and root diameter compared to root length (r=0.99 and 0.95, p<0.001) suggested that root diameter might be a more substantial yield attribute for determining the total dry root yield of *S. costus*.

Although 25 chemical compounds were found in the essential oil, the correlation matrix has been constructed amongst the 17 major constituents. The analyzed data revealed that caryophyllene oxide and eremanthin showed a strong and negative correlation with geranyl acetone (r=-0.65, p<0.01), germacrene B (r=-0.76 and r=-0.71, respectively, p<0.001) and dihydro-α-ionone (r=-0.47, p<0.05 and r=-0.61, p<0.01, respectively); however, former one also showed a negative correlation with α-costol (r=-0.72, p<0.001) and β-elemene (r=-0.59, p<0.01). The results illustrated that germacrene B was strongly and positively correlated with geranyl acetone with r value=0.90 (p<0.001). In addition, (E)-β- ionone exhibited a positive correlation with ar-curcumene (r= 0.54, p<0.05) and negative correlation with spathulenol (r= -0.5, p<0.05), dihydro-α-ionone (r= -0.53, p<0.05), germacrene B (r= -0.56, p<0.05) and geranyl acetone (r= -0.51, p<0.05).

**Figure 6:**
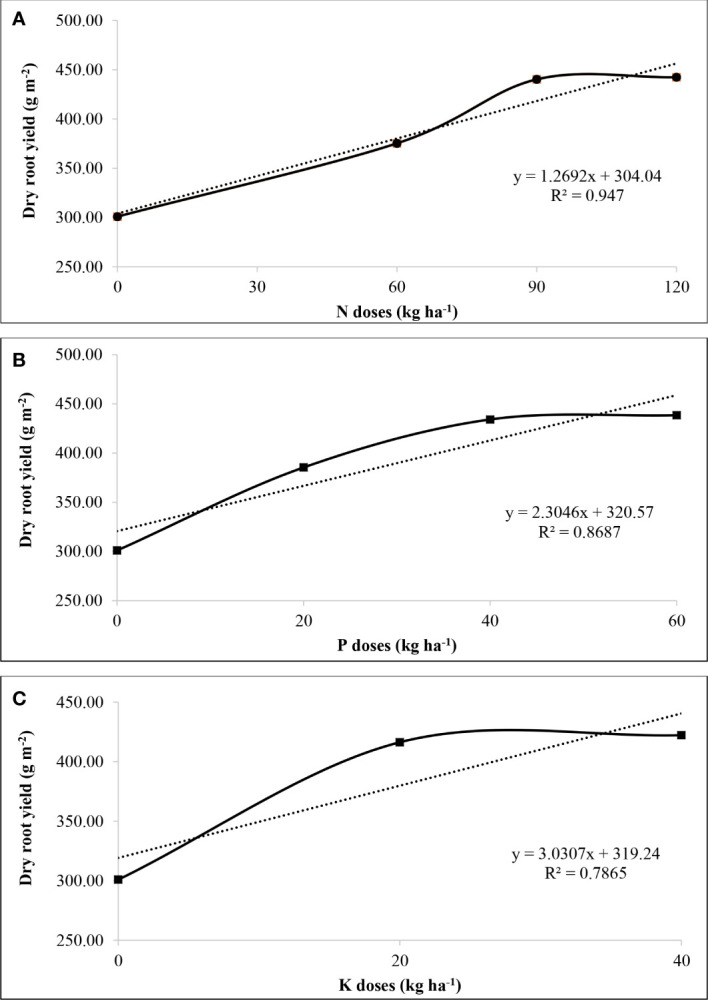
Regression equation developed between dry root yield, and N levels **(A)**, P levels **(B)**, and K levels **(C)**. The X-axis displays N, P and K levels in their respective graphs, and Y-axis represents the dry root yield in all three graphs.

Regression equations among N, P and K levels (independent variables), and dry root yield (dependent variables), were also studied (**Figure 6**). Among N, P, and K levels, dry root yield enhanced till 90 kg N ha^-1^, 40 kg P ha^−1^ and 20 kg K ha^−1^, respectively, with a constant value thereafter. A strong relationship of fertilizer levels, *i.e.*, N, P, and K with dry root yield, was observed by using linear equations y=12.69x + 3040.4 (r^2 ^= 0.947), y=23.04x + 3205.7 (r^2 ^= 0.868) and y=30.30x + 3192.4 (r^2 ^= 0.786), respectively.

### 3.9 Soil nutrient status after crop harvest

The analyzed data showed a non-significant effect of different levels of N, P, and K on soil pH and OC, however; a significant impact of fertilizer application on pH was observed over control. Besides this, N level had a significant impact on OC content. The results align with the previous outcomes of Begum et al. (2021), who reported increased OC content with higher levels of N fertilization.

After the harvest of *S. costus* roots, a significant effect of fertilizer application over control on major soil nutrients was observed (**Table 4**). Available N and P content increased with the rise in the dose of N and P. The highest available N (391.23 and 382.42 kg ha^-1^) and P (38.37 and 37.93 kg ha^-1^) were registered with N_120_ and P_60_ among levels of N and P, respectively. In contrast, levels of K didn’t show any significant variation in available N and P. However, available K was significantly affected by varying levels of nutrients and, the highest value was recorded with N_120_ (175.86 kg ha^-1^), P_60_ (174.62 kg ha^-1^), and K_40_ (176.54 kg ha^-1^) among their respective levels. In addition, the fertilizer additions recorded significantly higher available N (569.89 kg ha^-1^), P (55.69 kg ha^-1^), and K (251.42 kg ha^-1^) content after crop harvest over control (357.06 kg N ha^-1^, 33.16 kg P ha^-1^ and 129.33 kg K ha^-1^). The results corroborate the conclusions of Dong et al. (2012), who reported a notable influence of fertilizer applications on available nutrients in paddy soils.

**Table 4:** Effect of N, P, and K doses on soil pH, OC (%), available N, P and K (kg ha^-1^) after harvest of *S. costus*.

Treatments	pH	OC (%)	Available N (kg ha^-1^)	Available P (kg ha^-1^)	Available K (kg ha^-1^)
Nitrogen Level (kg ha^-1^)					
N_60_	6.64	0.67^b^	370.20^c^	36.15^c^	158.02^c^
N_90_	6.64	0.78^a^	378.34^b^	36.87^b^	168.96^b^
N_120_	6.62	0.81^a^	391.23^a^	38.37^a^	175.86^a^
SEm (±)	0.02	0.02	1.35	0.20	1.47
LSD (*P*=0.05)	NS	0.05	3.87	0.57	4.21
Phosphorus Level (kg ha^-1^)					
P_20_	6.66	0.72	377.44^b^	36.42^c^	161.44^c^
P_40_	6.64	0.76	379.92^b^	37.03^b^	166.78^b^
P_60_	6.61	0.77	382.42^a^	37.93^a^	174.62^a^
SEm(±)	0.02	0.02	1.35	0.20	1.47
LSD (*P*=0.05)	NS	NS	3.87	0.57	4.21
Potassium Level (kg ha^-1^)					
K_20_	6.64	0.74	379.15	37.03	158.68^a^
K_40_	6.63	0.75	380.69	37.23	176.54^b^
SEm(±)	0.01	0.01	1.10	0.16	1.20
LSD (*P*=0.05)	NS	NS	NS	NS	3.44
Control	6.87^b^	0.66	357.06^b^	33.16^b^	129.33^b^
Others	9.95^a^	1.12	569.89^a^	55.69^a^	251.42^a^
SEm(±)	0.03	0.03	2.40	0.35	2.61
LSD (*P*=0.05)	0.09	NS	6.88	1.02	7.50

SEm indicates standard error of mean; LSD indicates Least Significant Difference; NS indicates not significant at P = 0.05.

N60, N90, N120, represents N levels @ 60, 90, and, 120 kg ha-1, respectively; P20, P40 and P60, represents P levels @ 20, 40 and 60 kg ha-1, respectively; and K20 and K40 indicates K levels @ 20 and 40 kg ha-1, respectively.

## 4 Conclusions

Fertilizer applications of N, P, and K modulated the crop growth, dry root and essential oil yield, and chemical profile of *S. costus *essential oil. Among different levels of N, P, and K, the highest level of N (N_120_), P (P_60_), and K (K_40_) produced the tallest plant, the maximum number of leaves per plant, increased dry matter per plant, higher leaf length & width, and root length & diameter, followed by N_90_, P_40_, and K_20_. Similarly, the highest dry root yield was obtained by applying N_120_, P_60_, and K_40_ (4423.41, 4383.91, and 4222.34 kg ha^-1^, respectively), which produced at par results with N_90_, P_40_, and K_20_ (4401.87, 4340.92 and 4163.22 kg ha^-1^, respectively). The overall fertilizers applications increased the dry root yield and essential oil yield by 108.9% and 210.3%, respectively, over control. The fertilizer application caused significant variations in the chemical makeup of the root essential oil. The regression curve showed that dry root yield increased till the application of N_90_, P_40_, and K_20_ among varying levels of N, P, and K, after that, increased marginally. Hence, from the present study, we concluded that economically sustainable cultivation and *ex-situ *conservation of *S. costus* could be achieved by applying N_90_, P_40_, and K_20_ doses of fertilizer. Further studies are desired to understand the influence of other factors, especially micronutrients, on crop growth, productivity, and *S. costus* essential oil composition.

## Data availability statement

The raw data supporting the conclusions of this article will be made available by the authors, without undue reservation.

## Author contributions

SV: Manuscript writing. DD: Data collection. SB: Data collection. SatS: Statistical analysis. MK: Chemical profiling. AK: Manuscript editing. DK: Chemical profiling. SanS: Planting material. RC: Conceptualisation, monitoring, statistical analysis, review and editing. All authors contributed to the article and approved the submitted version.
